# Hemorrhage Detection and Segmentation in Traumatic Pelvic Injuries

**DOI:** 10.1155/2012/898430

**Published:** 2012-07-19

**Authors:** Pavani Davuluri, Jie Wu, Yang Tang, Charles H. Cockrell, Kevin R. Ward, Kayvan Najarian, Rosalyn H. Hargraves

**Affiliations:** ^1^Department of Electrical and Computer Engineering, Virginia Commonwealth University, Richmond, VA 23284, USA; ^2^Department of Computer Science, Virginia Commonwealth University, Richmond, VA 23284, USA; ^3^Department of Radiology, Virginia Commonwealth University, Richmond, VA 23298, USA; ^4^Department of Emergency Medicine, Virginia Commonwealth University, Richmond, VA 23298, USA; ^5^Virginia Commonwealth University Reanimation and Engineering Science Center (VCURES), Richmond, VA 23298, USA

## Abstract

Automated hemorrhage detection and segmentation in traumatic pelvic injuries is vital for fast and accurate treatment decision making. Hemorrhage is the main cause of deaths in patients within first 24 hours after the injury. It is very time consuming for physicians to analyze all Computed Tomography (CT) images manually. As time is crucial in emergence medicine, analyzing medical images manually delays the decision-making process. Automated hemorrhage detection and segmentation can significantly help physicians to analyze these images and make fast and accurate decisions. Hemorrhage segmentation is a crucial step in the accurate diagnosis and treatment decision-making process. This paper presents a novel rule-based hemorrhage segmentation technique that utilizes pelvic anatomical information to segment hemorrhage accurately. An evaluation measure is used to quantify the accuracy of hemorrhage segmentation. The results show that the proposed method is able to segment hemorrhage very well, and the results are promising.

## 1. Introduction

Hemorrhage is the leading cause of death in patients with traumatic pelvic fractures. These fractures are most often associated with motor vehicle accidents, falling from heights, and with crush injuries. The mortality rate for pelvic fractures range from 5% to 15%, and the mortality rate for pelvic fracture patients with hemorrhagic shock ranges from 36% to 54% [1, 2]. The majority of deaths caused due to hemorrhage occur within the first 24 hours after the injury [1, 3]. Hence, it is very important to quickly and accurately identify the source of bleeding and control the hemorrhage in a very short period.

The bleeding sites in the pelvic region originate from the fractured bone, venuous plexus, major pelvic veins, and/or damaged arteries [4, 5]. In recent years, contrast-enhanced computed tomography (CT) has been widely used by the radiologists for the examination of hemorrhage and characterization of fractures in traumatic pelvic injuries [2–4, 6]. However, depending on the CT slice thickness, it is rather time consuming for the radiologists to examine all the images, and it is often difficult to identify bleeding sites in the first review of these images. As time is a crucial factor in emergency medicine, there is a need for automated detection of hemorrhage. Identification of the bleeding site alone is not sufficient to assess the bleeding severity. Therefore, it is valuable to segment the detected hemorrhage to see if angiography is needed or not.

Detection and segmentation of hemorrhage in the pelvic region is very challenging due to the injury severity, variation in bleeding contrast from patient to patient, variation in size and shape of the bone, and the presence of several arteries in the region that may be injured. Due to the location of bones and arteries in various locations within the image, the entire image must be searched for hemorrhage. In addition, hemorrhage cannot be characterized by a single gray level. The gray levels of hemorrhage depend on the phase of CT scan. In the arterial phase (phase in which the pelvic region is scanned soon after the injection of contrast enhancer), the arteries in pelvic region are highlighted and if any hemorrhage is present, it is also differentiable from the soft tissues due to the contrast enhancer. But in the veinal phase (phase in which the pelvic region is scanned with some delay after the injection of contrast enhancer), the hemorrhage is not much differentiable from the soft tissues as the soft tissues start absorbing the enhancer. In general, the hemorrhage gray levels vary from patient to patient in a way that if a patient is bleeding heavily then the hemorrhage is highlighted more than in the patient where the bleeding is slow. Identification of hemorrhage boundary is not easy as the variation in gray level between the hemorrhage and the soft tissues does not vary much. Also, the hemorrhage gray level is not constant throughout the region. The gray level of hemorrhage is much higher around the center of the hemorrhage and fades out around the edges. Another important challenge is, the hemorrhage can occur due to the fractured bones. Hence, it is important to segment the hemorrhage region accurately when near bone. To overcome these challenges, anatomical information must be incorporated in the segmentation process.

Very few researchers have developed techniques for hemorrhage segmentation in the pelvic region [7]. Previous studies utilized a threshold-based method to segment hemorrhage. Furthermore, the method is only able to segment hemorrhage located in one particular region in the image. Even though there are very few studies on hemorrhage segmentation in pelvic region, there are several studies on medical image segmentation for various applications such as vascular segmentation, bone segmentation, hemorrhage segmentation, and so forth [8, 9]. Some of the existing methods are threshold based methods, region growing methods, clustering, markov random field (MRF) models, artificial neural networks, deformable models, atlas-based methods, level set methods, and so forth.

Threshold-based methods are one of the simplest methods that are used for segmentation. In this method, the pixels in the image are classified into groups based on a threshold value. Though this method is simple, it is sensitive to noise and intensity inhomogeneities, as it does not account for spatial characteristics of an image [10, 11]. Region-growing techniques are used to segment regions based on some similarity criteria. In this technique, a single seed is selected initially, and all the pixels around it are selected based on some predefined criteria. The limitation of this method is that it is susceptible to noise and partial volume effects [12, 13]. Clustering techniques like fuzzy *c*-means algorithms, *K*-means clustering, Kernel based methods, and so forth are unsupervised techniques developed for segmentation [14]. Though these techniques are computationally fast, they are either sensitive to noise or intensity inhomogeneities as they do not consider spatial context or depends on initialization.

Some researchers have used artificial neural networks for the segmentation [15, 16]. Artificial neural networks are parallel networks of processing elements that simulate biological learning. These networks have high-parallel ability and high interaction among the processing units enabling it to model any kind of process. However, these networks need to be trained beforehand, and the amount of time taken for training may be very long, and the results of these networks are influenced by initialization.

Deformable model techniques are other techniques that are used for segmentation [17, 18]. These techniques use closed parametric curves or surfaces that deform under the influence of internal and external forces. These techniques incorporate a smoothness constraint that provides robustness to noise and spurious edges. However, the disadvantages include poor convergence to concave boundaries and sensitivity to initialization. Level-set methods are other techniques that are based on a moving contour as the zero-level set of a time-evolving scalar function over a regular grid [19, 20]. The curve is deformed according to a given set of partial differential equations. Atlas-based methods are based on a standard template or atlas [21, 22]. The atlas is created based on the information of the anatomy that requires segmentation. The created atlas is then used as a reference for segmenting new images. The atlas-based methods are useful only for the segmentation of structures that do not exhibit great variation and are not extremely detailed.

Along with these segmentation techniques, there are other techniques such as watershed techniques that use concepts from edge detection and mathematical morphology to partition image into homogeneous regions [23]. These techniques suffer from over segmentation. However, recent studies have developed improved methods to overcome some of the drawbacks to segmentation [24, 25].

Some of these above mentioned techniques use a specific criterion to segment regions which are not usually adaptable to images with poor quality. However, incorporation of anatomical information makes the approach more adaptable to each and every image as the gray levels vary from image to image within the same patient. This paper presents a novel heuristic approach to segment hemorrhage which utilizes artery and bone information to initially detect the hemorrhage and then segments hemorrhage in multistages through hemorrhage matching, rule optimization, and region growing.

The rest of the paper is organized as follows.  Section 2 describes the methodology used for the study. The results section gives the results obtained using the described methods along with the data used for the study. This section also discusses the obtained results. Finally, the conclusion summarizes the work done and presents the future work for the study.

## 2. Methods

**Figure 1:**
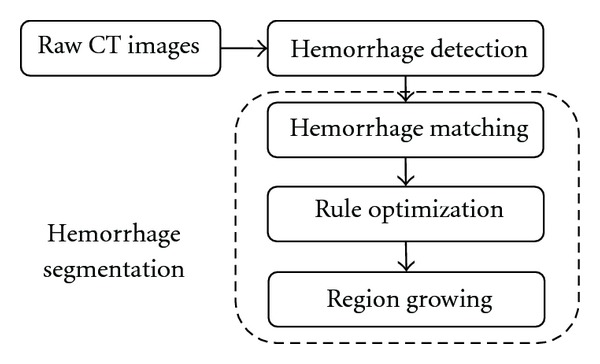
Schematic diagram of hemorrhage detection and segmentation.

Automated detection of the presence and extent of hemorrhage is extremely important for assessing injury severity and for fast accurate decision making and treatment planning. Hence, it is very crucial to utilize the artery and bone information in order to detect and segment the hemorrhage.  Figure 1 provides the schematic diagram of hemorrhage detection and segmentation.

The proposed hemorrhage segmentation technique involves locating the hemorrhage, hemorrhage matching, support vector machine (SVM) based rule optimization for determining hemorrhage regions under different cases, and finally region growing to determine the hemorrhage pixels missed even after the optimization. Each step in the process is explained in detail in the following subsections.

### 2.1. Hemorrhage Detection

**Figure 2:**
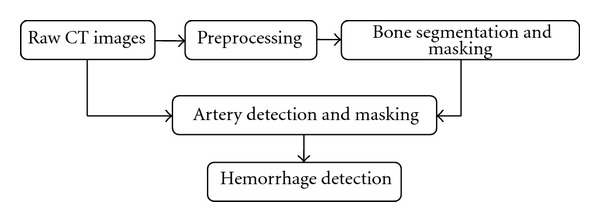
Schematic setup for hemorrhage detection.

Hemorrhage detection is vital in pelvic trauma to assess the injury severity and is the preparation step for hemorrhage segmentation. Our previous work focused on the hemorrhage detection from pelvic CT images [26, 27]. This work is a continuation of our previous work on hemorrhage detection.  Figure 2 shows the schematic setup for hemorrhage detection. A brief description of our previous work is provided below.

#### 2.1.1. Preprocessing

The first step in the hemorrhage detection is to remove any artifacts such as tables, hands, cables, and so forth from the pelvic CT images and extract the pelvic region. This is achieved using morphologic operations and blob analysis [26]. The next stage of hemorrhage detection is to segment bone.

#### 2.1.2. Bone Segmentation and Masking

**Figure 3:**
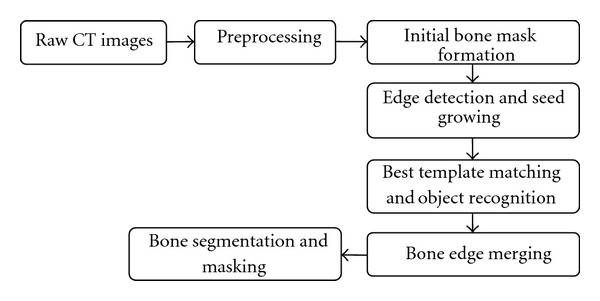
Bone segmentation setup.

Once the pelvic region is extracted, the pelvic bones are segmented.  Figure 3 below shows the setup for bone segmentation. This involves bone mask formation, edge detection, shape matching and object recognition, edge merging, bone segmentation, and masking. The bone mask is formed by setting a threshold in order to separate bone regions from nonbone regions. However, nonbone regions with gray levels greater than the threshold may also be determined as bone regions at this stage. These false bone regions are later eliminated in the shape matching and object recognition phase. Canny edge detection technique is used to determine the edges of the obtained mask. This technique is used because of its ability to detect true strong and weak edges. Once the bone edges are determined, seed growing technique is used to select pixels closer to the true edge of the bone region. This gives the initial segmented bone image. Later, shape matching is used to determine the best templates that match these segmented regions in each image. These templates are obtained from Visible Human Project dataset manually and offline. A total of 73 templates are used for the study. The best template detection helps determine the position of arteries in the pelvic region, explained later. This process eliminates the nonbone objects from the image by determining the shape matching cost [28–32]. Hence, initial bone regions are segmented.

After segmenting the bone regions, the edges of the bones are determined using canny edge detection technique. In some cases, the edges of the bones may not be fully connected. In order to ensure better masking of the bone, the edges of the bone in the current slice are merged with the bone in the previous and the next slice. Since the study is not about fracture detection, bone merging will have minimal effect on the hemorrhage detection. The next step is final bone segmentation. This is done in a way similar to that of the initial bone segmentation using seed growing technique. The final segmented bone is masked by setting its gray level values to zero.

#### 2.1.3. Artery Detection and Masking

The major arteries in the pelvic region are aorta and its branches (common iliac arteries). Since arteries and bleeding are of similar gray levels, the detection of arteries will help estimate the bleeding gray levels. Hence, the next step is to detect arteries in the pelvic region. The aorta, common iliac arteries, and the external iliac arteries are determined using template matching and from segmented bone location [26, 29–31, 33]. The internal iliac arteries are determined from the position of the external iliac arteries. These detected arteries are then masked to avoid any false hemorrhage detection.

#### 2.1.4. Hemorrhage Detection

After masking the major arteries, the image is searched for unwanted objects other than hemorrhage. The unwanted objects are residual bone pixels or any pixels that are left even after masking the bone and arteries other than the hemorrhage pixels. They are removed by using morphologic operations. After the filtration of unwanted objects, the region in the image that falls within the gray-level range of arteries is considered as hemorrhage and its center coordinates are identified as the centroid of the hemorrhage region [26, 30].

The hemorrhage detected may not be the complete region of hemorrhage especially during the veinal phase. If some of the hemorrhage pixels gray levels are similar to that of soft tissues, especially during the veinal phase, then those pixels would have been eliminated during the filtration of unwanted objects. In addition, the gray levels of hemorrhage that lie within artery gray levels and higher are considered as hemorrhage. However to identify the hemorrhage severity, the entire hemorrhage region must be known.

### 2.2. Hemorrhage Segmentation

Another important challenge is the identification of bleeding next to the bone, as the hemorrhage can occur due to the fractured bones. Hence, it is important to segment the hemorrhage region accurately when present next to the bone. The proposed segmentation process consists of hemorrhage matching, rule optimization, and region growing, which are described in detail in the following sub sections.

#### 2.2.1. Hemorrhage Matching by Mutual Information Maximization

The first step of hemorrhage segmentation is hemorrhage matching. The hemorrhage region detected using the previously mentioned method does not contain all the hemorrhage pixels especially the boundaries of the hemorrhage. Hemorrhage matching helps identify the threshold, that is, the optimum minimum gray level *G*_opt_ for segmenting the hemorrhage region. This is accomplished using the mutual information maximization (MIM). First, a window of size *q* × *q* in the preprocessed CT image is selected as a region of interest (ROI) *S* around the centroid of the detected hemorrhage. The range [*G*_min⁡_, *G*_max⁡_] of the hemorrhage gray levels are then determined from the detected hemorrhage. Then a gray level *G*_mi_, where *G*_min⁡_ ≤ *G*_mi_ ≤ *G*_max⁡_ is chosen as the minimum gray level and all the pixels in ROI *S* that lie within [*G*_mi_, *G*_max⁡_] are chosen as hemorrhage pixels. Morphologic operations are performed to eliminate any nonhemorrhage regions in each of these determined hemorrhage images. This obtained hemorrhage image is individually compared to the initial detected hemorrhage image using mutual information (MI) technique in order to find the amount of information each image contains about the detected hemorrhage [34]. This MI is calculated between the previously detected hemorrhage image and the hemorrhage images obtained for different gray level ranges. The cut-off gray level that contains the maximum information about the detected hemorrhage is considered as the optimum minimum gray level *G*_opt_ at this stage. The mutual information in this process is determined in the following manner. Let *C*_*d*_ be the detected hemorrhage image from the previous section, and let {*B*_1_,…, *B*_*i*_,…, *B*_*m*_}, where *i* = 1,2,…, *m* be the hemorrhage regions obtained with the initial cutoff that ranges within [*G*_min⁡_, *G*_max⁡_]. The mutual information between images *C*_*d*_ and *B*_*i*_ is determined using

(1) $$\begin{array}{c} \text{MI}\;\left(C_{d},B_{i}\right)=H\;\left(C_{d}\right)+H\;\left(B_{i}\right)-H\;\left(C_{d},B_{i}\right), \end{array}$$

where *H*(*C*_*d*_), and *H*(*B*_*i*_), are the entropies of images *C*_*d*_ and *B*_*i*_, and *H*(*C*_*d*_, *B*_*i*_) is their joint entropy, and are computed as follows:

(2) H  (Cd)=−  ∑cPCd(c)log⁡⁡PCd(c),H  (B  i)=−  ∑b  PBi(b)log⁡  PBi  (b),H  (Cd,Bi)=−  ∑c,b  PCd,Bi(c,b)  log⁡  PCd,Bi  (c,b),

where, *P*_*C*_*d*__(*c*), *P*_*B*_*i*__(*b*) denote individual probability distributions. *P*_*C*_*d*_,*B*_*i*__(*c*, *b*) denotes the joint probability distribution of the images.

The cut-off gray level for which the mutual information between *C*_*d*_ and *B*_*i*_ is maximum, is the optimum gray level *G*_opt_ and the image is the optimum image at this stage of segmentation. This process is called mutual information maximization. The pixels within the image that lie within [*G*_opt_, *G*_max⁡_] are considered as hemorrhage pixels, and *G*_opt_ is considered as the minimum hemorrhage gray level from now on. However, *G*_opt_ may not be the actual minimum gray level of hemorrhage as these cut-off gray levels are from the detected hemorrhage and may not include all the hemorrhage pixels such as boundary pixels which might have gray levels less than *G*_opt_. From now on, the hemorrhage region is denoted by *R*. These undetermined hemorrhage pixels are segmented using the method explained in the following subsection.

#### 2.2.2. Support Vector Machine-Based Rule Optimization for Hemorrhage Segmentation

The utilization of pixel gray levels alone is not enough to determine whether a pixel is hemorrhage or not. Hence, there is a need for incorporation of pixel information such as location, gradient, and so forth around the detected hemorrhage region to properly classify hemorrhage pixels from the nonhemorrhage pixels. This incorporation must be adaptable depending on whether the hemorrhage pixel is in the neighborhood of all hemorrhage pixels or soft tissue pixels. This study incorporates pixel gray levels, distance of the pixel from the hemorrhage foci (the pixel with maximum gray level), the gray level variation within the selected window, and the magnitude of the gradient of each pixel within the selected window in order to achieve better segmentation.

**(1) Rule Generation**

Let *B*_opt_ be the hemorrhage region image obtained using MIM technique. Let *T*_opt_ be the boundary of the hemorrhage region in image *B*_opt_ and *p*(*x*_*i*_, *y*_*j*_) be the hemorrhage pixel of *T*_opt_. A window *W* of size *m* × *m*  (*m* < *q*) is selected around pixel *p*(*x*_*i*_, *y*_*j*_). There are three cases that need to be considered for an optimum segmentation: (1) the selected window *W* contains all hemorrhage pixels with gray levels within [*G*_opt_, *G*_max⁡_], (2) the majority of the pixels in *W* being hemorrhage pixels and with gray levels ≥*G*_opt_, and (3) the majority of the pixels in *W* (being hemorrhage or soft tissue pixels) with gray levels <*G*_opt_. Therefore, heuristic rules need to be generated for each case in order to optimally segment hemorrhage from nonhemorrhage pixels. The rule for each case is given as follows.

**Case 1**

*W* containing all hemorrhage pixels with gray levels within [*G*_opt_, *G*_max⁡_].

If the window contains all pixels with gray levels within [*G*_opt_, *G*_max⁡_], then all these pixels are hemorrhage pixels and can be added to the hemorrhage region *R*. So the rule in this case is that the pixel must satisfy the below condition in order to be added to region *R*.

(3) $$\begin{array}{c} R=\left\{\text{pixel}:p_{\left(x_{r},y_{s}\right)}\;|\;G_{\text{opt}}\leq p\left(x_{r},y_{s}\right)\leq G_{\max}\right\} \end{array}$$

**Case 2**

*W* containing a majority of hemorrhage pixels, that is, more pixels with gray levels ≥*G*_opt_.

If the window contains a majority of (i.e., >50%) hemorrhage pixels with gray levels ≥*G*_opt_, then the probability of the rest of the pixels within the neighborhood being hemorrhage is high. As a result, the neighborhood will be dominant with hemorrhage pixels. As the neighborhood is dominant with hemorrhage pixels, pixel gray level and the distance of the pixel from the foci are incorporated into the rule in this case. These parameters are only considered because the variation in magnitude of the gradient and the variation between the pixel gray levels will not add any advantage in differentiating hemorrhage pixels from soft tissue pixels. Each of the parameters used will have a certain weightage which needs to be incorporated for determining hemorrhage pixels. Therefore, the rule is if the pixel satisfies the condition given in (4), then it is considered as hemorrhage pixel and is added to region *R*.

(4) R={pixel:p(xr,ys) ∣ w1×p(xr,ys)  +w2×D(xr,ys)+b>0},

where *D*(*x*_*r*_, *y*_*s*_) is the distance between the pixel in the window and the foci (*x*_*f*_, *y*_*g*_), and is given by

(5) $$\begin{array}{c} D\left(x_{r},y_{s}\right)=\sqrt{{\left(x_{f}-x_{r}\right)}^{2}+{\left(y_{g}-y_{s}\right)}^{2}} \end{array}$$

and *w*_1_ and *w*_2_ are the weights and *b* is the bias.

In order to achieve proper segmentation, these weights need to be optimized. An SVM-based dual Lagrangian technique is used to determine the optimized weights and bias. This optimization technique is explained in the later subsections.

**Case 3**

*W* containing a majority of pixels (soft tissue or hemorrhage) with gray levels <*G*_opt_.

If the window contains more (i.e., >50%) pixels (soft tissue or hemorrhage) with gray levels <*G*_opt_, then the probability of the rest of the pixels within the neighborhood being hemorrhage is lower. Hence, it is required for the algorithm to be more restrictive in this case when compared to the other two cases. Hence, inclusion of magnitude of gradient and the gray level variation within the window along with the pixel gray level and its distance from the foci will help avoid oversegmentation which is crucial. Therefore, the rule associated with this case is

(6) R={pixel:p(xr,ys) ∣ w3×p(xr,ys)+w4×D(xr,ys)  +w5×V(xr,ys)+w6  ×|∇f(xr,ys)  |+b1>0},

where,

(7) $$\begin{array}{c} V\left(x_{r},y_{s}\right)=p\left(x_{b},y_{b}\right)-p\left(x_{r},y_{s}\right), \end{array}$$

where *p*(*x*_*b*_, *y*_*b*_) is the gray level of the center coordinate of window *W*, *V*(*x*_*r*_, *y*_*s*_) is the difference in gray level of the center coordinate and the gray level of the pixel in the window. The magnitude of the gradient of each pixel is given in

(8) $$\begin{array}{c} \left|\nabla f_{\left(x_{r},y_{s}\right)}\right|=\sqrt{{\left(\frac{\partial f}{\partial x_{r}}\right)}^{2}+{\left(\frac{\partial f}{\partial y_{s}}\right)}^{2}}. \end{array}$$

If a pixel in the selected window satisfies the above mentioned condition, then it is considered as hemorrhage and is added to the existing hemorrhage region *R*.

The weightage of the parameters given in (6) must be determined for each image as these can vary among different images. The weights *w*_3_ through *w*_6_ and the bias *b*_1_ are later optimized using SVM-based dual Lagrangian optimization technique.

**(2) SVM Based Rule Optimization**

The weights used in the previously mentioned rules must be optimized to ensure proper segmentation. These weights must be optimized for each image as these can vary from image to image within the same patient. An SVM-based Lagrangian function in the dual space is used to optimize the weights and the bias. The optimization is solved by the saddle point of Lagrange function in the dual space. For optimization, the data for soft tissue pixels is selected outside the boundary *T*_opt_, and the data for hemorrhage pixels is selected from the pixels within the boundary. The selection of these pixels outside the boundary and within the boundary will facilitate the process of identifying the gray level of the boundary pixels. A tenfold cross-validation is used for training and testing the data in order to determine the optimum weights and bias for each of the parameters used in the study. The size of the data set for training and testing depends on the size of the boundary of the hemorrhage in each image. The weights and the bias are optimized separately for each case. For solving with the Lagrangian in dual space, Karush-Kuhn Tucker conditions for the optimum of a constraint function are considered in the study [35].

With those conditions, the dual Lagrangian is given as follows:

(9) $$\begin{array}{c} L_{d}\left(\alpha \right)=\sum_{i=1}^{n}\alpha_{i}-\frac{1}{2}\sum_{i,j=1}^{n}y_{i}y_{j}\alpha_{i}\alpha_{j}x_{i}x_{j}, \end{array}$$

where, *α*_*i*_ are the Lagrange multipliers, and *x* and *y* are the inputs and the labels and *n* is the dimensionality of the input.

The inputs in this study are pixel gray level, distance of pixel from the foci, magnitude of the gradient, and the gray level variation. If it is Case 2, there are only 2 input variables. The labels are the classes. In this study, there are two classes: hemorrhage and nonhemorrhage class.

This standard quadratic optimization problem is expressed in matrix notation and formulated as follows:

(10) Maximize Ld(α)=−0.5αTHα−1Tα, subject  to yTα=0, 0≤α≤C,  

where *H* is the Hessian matrix (*H*_*ij*_ = *y*_*i*_ *y*_*j*_ *x*_*i*_ *x*_*j*_), *C* is the penalty parameter, and 1 is a unit vector 1 = [1 1 ⋯ 1]^*T*^. *C* is chosen as the upper bound of *α* because with *C* the influence of training data points that remain on the “wrong” side of a separating nonlinear hypersurface is limited. Also, the width of the soft margin is controlled by a corresponding *C*. Large *C* leads to small number of misclassifications, smaller margin and vice versa. In our study, *C* is considered to be greater than zero and less than infinity for feasibility. The penalty parameter is optimized using 10-fold cross-validation technique. Solution *α*_0_ from the above equation determines the parameters of the optimal hyperplane *w*_0_ and *b*_0_ as given in

(11) $$\begin{array}{c} w_{0}=\sum_{i=1}^{N_{\text{sv}}}\alpha_{0i}y_{i}x_{i},\\ b_{0}=\frac{1}{N_{\text{fsv}}}\left(\sum_{s=1}^{N_{\text{fsv}}}\left(\frac{1}{y_{s}}-x_{s}^{T}w_{0}\right)\right), \end{array}$$

where *w*_0_ and *b*_0_ are the optimized weights and bias, *N*_sv_ denotes the number of support vectors, and *N*_fsv_ denotes the number of free support vectors.

In (11), the support vectors are only used because the Lagrange multipliers are zero for nonsupport vectors. Finally, with the optimal weights and bias, the decision hyperplane *d*(*x*) is determined using

(12) $$\begin{array}{c} d\left(x\right)=\sum_{i=1}^{n}w_{\text{0}i}x+b_{0}, \end{array}$$

where *x* is the test data.

The output of the test data is determined by using an indicator function given in

(13) $$\begin{array}{c} i_{F}=\mathrm{sign}\left(d\left(x\right)\right). \end{array}$$

The number of wrongly classified pixels are determined by comparing the test output with the desired output. The obtained optimized weights and bias are used to determine if a pixel is a hemorrhage pixel or not. The optimized weights are used in the rules, and the pixels in each window *W* are considered as hemorrhage if they satisfy the optimized rules. However, there is a slight chance of missing the hemorrhage pixels which are outside the boundary and are not located in the selected window. Hence, it is required to include these pixels in the hemorrhage region. Region growing process is used to grow the region around the already determined hemorrhage region *R* to determine any hemorrhage pixels that are missed during the optimization process. This is described in the following subsection.

#### 2.2.3. Region Growing

**Figure 4:**
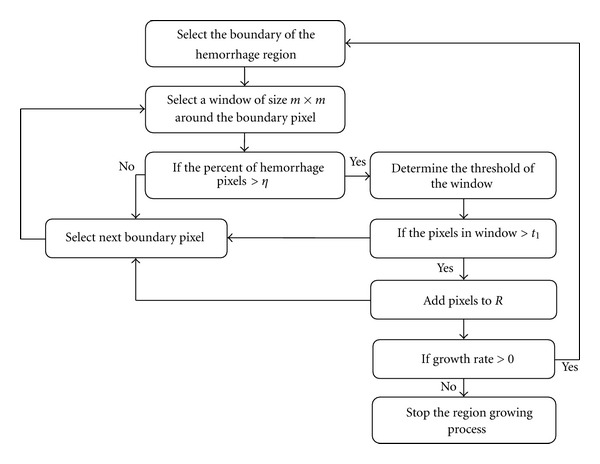
Region growing process.

The region growing process is the final phase of hemorrhage segmentation. This process is used to determine any missed hemorrhage pixels that are located outside the boundary of *R*.  Figure 4 shows the region growing process used in this study. The region growing process consists of several steps. First, the boundary of the segmented hemorrhage *R* from the previous phase is used to select a window of size *m* × *m* around each boundary pixel. If the percent of total number of pixels within that window that satisfy the conditions described earlier are >*η*, the pixel factor, then the threshold *t*_1_ for the window is determined using

(14) $$\begin{array}{c} t_{1}=\text{m}{\text{e}}_{1}+\text{st}{\text{d}}_{1}, \end{array}$$

where me_1_ and std_1_ are the mean and standard deviation of the gray levels of all the nonbackground pixels in the window and are given by

(15) $$\begin{array}{c} \text{m}{\text{e}}_{1}=\frac{\sum_{x=1}^{m}\sum_{y=1}^{m}f\left(x,y\right)\;\;}{m\times m-\text{Card}\left(S\right)},\\ \text{st}{\text{d}}_{1}=\sqrt{\frac{\sum_{x=1}^{m}\sum_{y=1}^{m}{\left(f\left(x,y\right)-m_{1}\right)}^{2}\;}{m\times m-\text{Card}\left(S\right)},} \end{array}$$

and *S* = {(*x*, *y*) | *f*(*x*, *y*) = 0} is the set of pixels located in the background having zero gray level. Card(*S*) denotes the cardinality of set *S*.

If any of the pixels that lie outside the boundary and within the window satisfy *t*_1_, then they are considered as hemorrhage pixels and are added to the existing hemorrhage region *R*. This entire region growing process is repeated for all the boundary pixels. This complete process constitutes one epoch. If the growth rate of hemorrhage region is >0 in the current epoch, then the entire process is repeated starting from selecting the boundary of the hemorrhage region, else the region growing process is stopped. The growth rate in each epoch is calculated using

(16) $$\begin{array}{c} \text{Growth}\;\;\text{rate}=\frac{E_{c}-E_{p}}{E_{c}}\times 100, \end{array}$$

where *E*_*c*_ is the total area of the hemorrhage by the end of current epoch, and *E*_*p*_ is the total area of the hemorrhage by the end of previous epoch. The total region-grown by the end of the region growing process is considered as the final segmented hemorrhage.

### 2.3. Evaluation Measure for Segmentation

Once the hemorrhage is segmented, a suitable measure is required to quantify the accuracy of segmentation. This study utilizes a measure called missegmented area. The missegmented area measure represents the uncommon area of segmented region (i.e., the pixels of segmented region that are not a true hemorrhage) compared to the gold standard area of segmented hemorrhage. If *A*_1_ and *A*_2_ are the areas of actual and the segmented region, the missegmented area of the two regions is defined as

(17) $$\begin{array}{c} \frac{\text{Cardinality}\;\;\left\{K\right\}}{\text{Cardinality}\;\;\left\{A_{1}\right\}}\times 100, \end{array}$$

where

(18) $$\begin{array}{c} K=\left\{\text{pixels}:p\;|\;p\in A_{1}\cup A_{2},p\notin A_{1}\cap A_{2}\right\}. \end{array}$$

Based on this measure, the segmented hemorrhage will be classified into three categories: good, acceptable, and unacceptable through consultation with a trauma physician and a radiologist, who identified actual hemorrhage contour as the ground truth.

The segmented regions with missegmented area <10% will be classified as good, and regions with missegmented area between 10% and 20% will be considered as acceptable, and finally any region with missegmented area greater than 20% will be considered as unacceptable. These ranges for good, acceptable, and unacceptable are used in the study based on the discussion with expert radiologists who utilize these ranges to determine if a region is properly segmented or not and how severe the bleeding is. The numerical values of *K* itself are not considered in this study as the radiologists are not concerned about the numerical values because these values do not provide any additional information to radiologists about the injury severity.

## 3. Results and Discussion

### 3.1. Dataset

The dataset for the study is obtained from Carolinas Health System and Virginia Commonwealth University Medical Center. The data is collected from twelve pelvic trauma patients with each scan consisting of 30 to 70 images with a total of 515 images. These twelve patients exhibit very mild to severe hemorrhage and these patients are selected at random. From the discussion with expert radiologists, it has been found that these number of images selected are sufficient to validate the performance of the proposed method. A statistical *t*-test is conducted in addition to see if the total number of images used in the study is statistically significant or not. A *P* value < 0.05 is considered as statistically significant, and a greater value is considered statistically not significant. These images chosen are axial CT images with 5 mm slice thickness. 

### 3.2. Results and Discussion

The proposed method is tested on twelve pelvic trauma patients who exhibit mild to severe bleeding. The total number of images used for the study from these twelve patients is 515 images. The dimensions of each image are 512 × 512 pixels. A *P* value of 0.0029 is obtained using the *t*-test showing that the selected number of images is statistically significant to test the proposed method. The CT scan include both images taken during arterial phase and the veinal phase. The hemorrhage is more distinguishable in the arterial phase than in the veinal phase. 

The ROI size *q* × *q* in the hemorrhage matching section is chosen as 100. This value is chosen because a smaller window size may not contain the entire hemorrhage region and if a larger size is chosen, then the nonhemorrhage tissues might be present along with the hemorrhage region making hemorrhage segmentation much complicated. During the rule optimization, the values chosen initially for the penalty parameter *C* are 0.1, 0.01, and 0.001. The optimal *C* value obtained is different for each image in the patient. It is dependent on the accuracy of classification. The penalty parameter for which the accuracy is maximum is chosen as the optimal penalty parameter. For the region growing process, the window size *m* is chosen as 3. The pixel factor *η* is chosen as 50. This value is selected because, in order for the algorithm to be restrictive in region growing, it is required to consider a window that is dominated by the hemorrhage pixels. If the value is chosen lower than this, the probability of oversegmentation might increase, and if the value is chosen higher than this value, then the algorithm becomes too restrictive and might leave hemorrhage pixels out affecting the segmentation.

**Figure 5:**
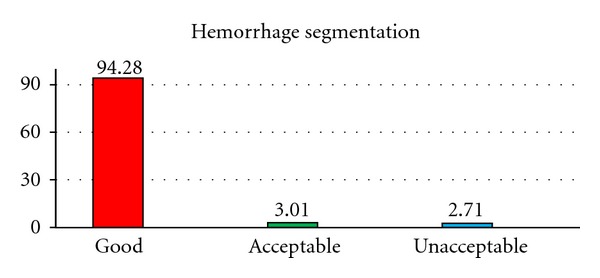
Proposed method performance for hemorrhage segmentation.

Figure 5 shows the hemorrhage segmentation results. The proposed method is able to segment the hemorrhage very well for 94.28% of the cases used in the study. These cases are considered as good as the missegemented area is <10%. The overall average missegmented area is 5.3% For 3.01% of the cases, the segmented hemorrhage is acceptable. The average missegmented area in these acceptable cases is 14.47%. For the remaining 2.71% of the cases, the segmented hemorrhage is unacceptable, and the average missegmented area is 26.52%.

**Figure 6:**
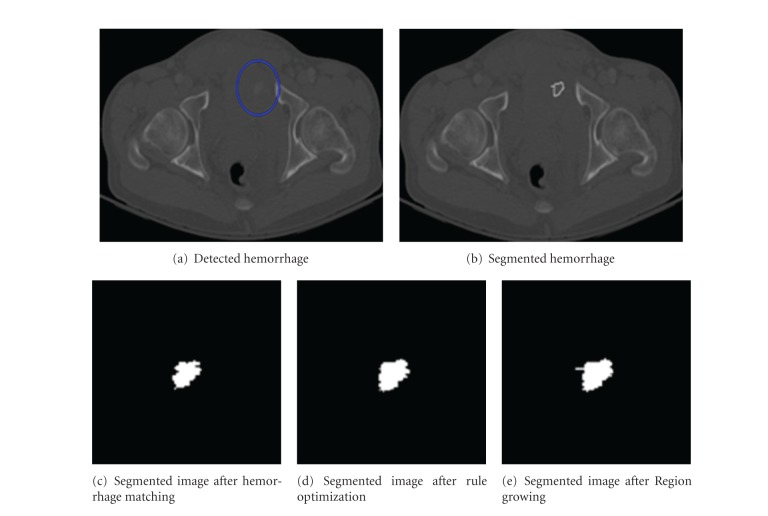
Sample hemorrhage segmentation results.

**Figure 7:**
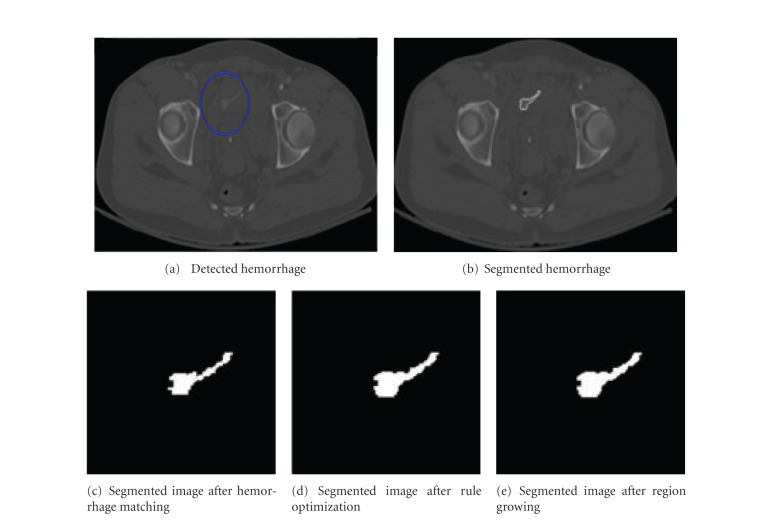
Sample hemorrhage segmentation results.

Figures 6 and 7 show the results of segmented hemorrhage. These are some of the cases where hemorrhage is very well segmented. The results show that the proposed method has segmented hemorrhage very well. Figures 6(c) through 6(e) gives the segmentation results at various stages of segmentation, that is, segmentation results after hemorrhage matching using MIM, rule optimization, and region growing. The percentile of hemorrhage area grown from the results of MIM technique to optimization technique is 24.6% and the percentile of hemorrhage area grown from the optimization to region growing is 3.53%. These results show that the rule optimization helps in determining hemorrhage accurately, and the region growing helps determine the missing hemorrhage pixels.

In the case of patient in Figure 7, the percentile of hemorrhage area grown from the results of MIM technique to optimization technique is 22.3% and the hemorrhage area is not grown during the region growing process as all the hemorrhage pixels are identified in the earlier stage itself.

**Figure 8:**
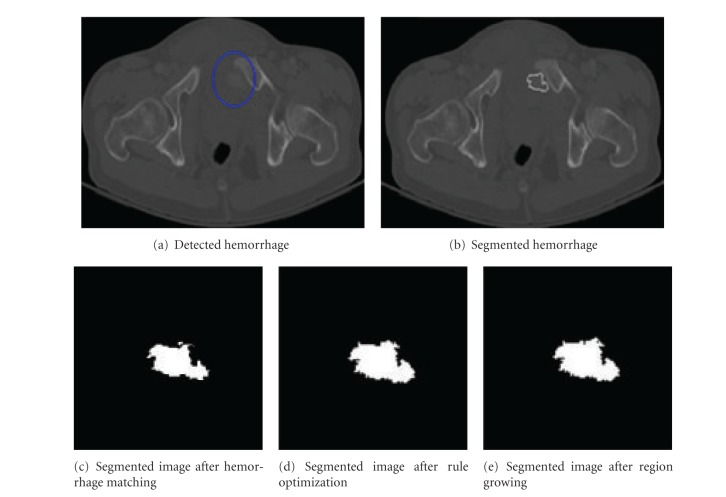
Sample segmentation results for hemorrhage located next to bone.

Figure 8 shows the segmentation results of hemorrhage located next to the bone. This segmentation is considered as acceptable. As hemorrhage is located next to the bone, the gray levels of the faded bone edges might be similar to hemorrhage gray levels. The use of distance information and gray level variation information helped in differentiating the hemorrhage from the bone regions for majority of the pixels. However for few pixels, the proposed method is unable to differentiate between the hemorrhage and bone pixels. Figures 8(c) through 8(e) shows the performance of proposed method at various stages. In these figures, the percentile of hemorrhage area grown from the results of MIM technique to optimization technique is 25.42%. And the percentile of hemorrhage area grown from the optimization to region growing is 0.56%. The hemorrhage area grown through region growing is much less in this case. It can be observed from this that the rule optimization has segmented most of the hemorrhage pixels.

The results are validated on the basis of assessment and evaluation made by the radiologists on the CT images. The proposed method is able to segment hemorrhage very well for majority of the cases. The segmentation is unacceptable in few cases which may be due to the bridging of hemorrhage pixels through soft tissue pixels. Hence, these few pixels are left out during the segmentation. Increasing the size of selected window might help segment these pixels. However, the tradeoff is, it might lead to oversegmentation. Incorporating pixel information into the rule optimization helps to differentiate the hemorrhage from soft tissue and bone region. The optimization technique is able to segment hemorrhage edges very well. The region growing process is able to determine the missed hemorrhage pixels. In addition, the proposed method is able to segment hemorrhage edges that may not be measurable through visual inspection. The overall processing time of hemorrhage detection and segmentation for each slice in a scan is a few seconds when run on a Intel(R)Core(TM)i7-2600 CPU@3.40 GHz machine. This is much faster than the manual hemorrhage detection that takes more than a minute for each slice. The entire process is fully automated. Automated detection with relatively high speed helps physicians make fast and accurate diagnostic decisions and treatment planning which is very crucial for traumatic pelvic injuries.

## 4. Conclusions and Future Work

This paper presents a fully automated hemorrhage segmentation technique that consists of hemorrhage matching, rule optimization, and region growing. These techniques incorporate the pixel gray level information, magnitude of the gradient, distance measure, and the gray level variation for segmentation. The results show that the proposed method is capable of segmenting hemorrhage well. Automated hemorrhage segmentation, once verified with more data, will be an important component of computer-assisted decision making system. Future work will focus on the quantitative measurement of hemorrhage such as determining hemorrhage volume, identifying the location of hemorrhage with respect to the bone, and so forth on the basis of larger data set.
